# Supplementary material for: Distinct Elevational Patterns and Their Linkages of Soil Bacteria and Plant Community in An Alpine Meadow of the Qinghai–Tibetan Plateau
Source: Microorganisms. 2022 May 19;10(5):1049. doi: 10.3390/microorganisms10051049 (PMC9143282; doi:10.3390/microorganisms10051049)
Supplement: Supplementary file 1 [file microorganisms-10-01049-s001.zip › microorganisms-1729523-supplementary.pdf]

Supplementary Materials

Distinct Elevational Patterns and Their Linkages of Soil Bacteria and Plant Community in An Alpine Meadow of the Qinghai–Tibetan Plateau

Jing Cong [1], Wei Cong [2], Hui Lu [2] and Yuguang Zhang [2,*]

[1] College of Marine Science and Biological Engineering, Qingdao University of Science Technology, Qingdao 266042, China; yqdh77@163.com

[2] Institute of Forest Ecology, Environment and Protection, Chinese Academy of Forestry and the Key Laboratory of Biological Conservation of State Forestry Administration, Beijing 100091, China; cong0915wei@163.com (W.C.); susanluhui@163.com (H.L.)

* Correspondence: yugzhang@sina.com.cn; Tel./Fax: +86-01062888594

Table S1. The climate and soil physicochemical characteristics in all six study sites

Environmental Factors	SJY-4790	SJY-4480	SJY-4140	SJY-3880	SJY-3490	SJY-3220
Mean annual temperature of the warmest quarter (°C)	4.90	6.20	6.30	7.40	10.00	11.60
Mean annual precipitation (mm)	417.00	386.00	372.00	354.00	344.00	319.00
Soil moisture (%)	40.42±5.74a	38.12±8.18a	28.88±4.44b	20.13±3.48c	17.84±3.81cd	14.34±5.41d
pH	6.21±0.30e	6.65±0.28d	7.38±0.12c	7.54±0.09b	7.72±0.07a	7.75±0.05a
Soil organic carbon (SOC) (g/kg)	79.25±15.21a	68.06±21.65b	62.57±12.17b	42.51±8.96c	25.09±3.76d	23.82±2.37d
Total nitrogen (TN) (g/kg)	6.47±1.33a	5.69±1.50b	5.67±0.73b	3.85±0.49c	2.61±0.35d	2.81±0.16d
SOC/ TN ratio	12.29±0.93a	11.82±1.12ab	10.99±.11b	11.12±2.45b	9.62±0.61c	8.47±0.49d
Total phosphorus (TP) (g/kg)	0.64±0.17ab	0.61±0.06ab	0.67±0.12a	0.58±0.05b	0.45±0.02c	0.51±0.03bc
Available N (mg/kg)	509.62±111.32a	463.62±132.59ab	410.03±61.73b	287.98±41.78c	204.04±40.43d	181.68±15.46d
Available P (mg/kg)	26.33±8.09b	18.81±3.29c	39.02±19.12a	29.14±5.96b	8.46±2.67d	20.67±3.22c
NH_4^+-N (mg/kg)	11.62±8.64a	11.20±8.38a	6.56±1.88b	5.54±1.57b	4.89±1.39b	5.87±1.85b
NO_3^--N (mg/kg)	61.49±31.98a	43.51±7.29b	53.54±11.54ab	36.98±10.10b	39.49±11.74b	31.61±13.38b

Data present the mean value and standard error. Significant differences among study sites are indicated by alphabetic letters. $P < 0.05$.

Table S2. Soil bacterial richness at the phylum level at six study sites

**Phylum**	**SJY-4790**	**SJY-4480**	**SJY-4140**	**SJY-3880**	**SJY-3490**	**SJY-3220**
Proteobacteria	1060.69±71.75d	1005.00±63.30cd	992.62±69.48c	863.54±81.81a	934.08±70.61b	883.69±103.45ab
Acidobacteria	618.69±86.85a	651.77±60.30a	625.77±80.78a	637.69±69.12a	735.00±42.90b	732.77±65.93b
Actinobacteria	367.62±36.72a	396.31±61.24a	567.54±79.67b	573.85±66.16b	623.77±63.79bc	651.69±141.29c
Verrucomicrobia	275.54±2.79c	208.69±18.35b	200.00±14.89b	175.23±26.25a	195.62±21.57b	177.08±20.75a
Bacteroidetes	357.23±46.67d	291.00±40.05c	264.62±46.88bc	238.5440.92ab	243.85±29.75ab	217.46±32.78a
Planctomycetes	125.08±18.43a	137.77±17.05a	162.08±16.25b	154.85±20.29b	179.38±18.51c	188.69±34.53c
Chloroflexi	51.92±6.06b	48.08±5.01ab	48.54±7.04ab	42.85±8.63a	60.38±10.70c	55.15±13.13bc
Firmicutes	55.62±8.95a	64.31±8.60b	82.69±9.29c	79.15±8.85c	96.15±11.43d	92.85±17.19d
Crenarchaeota	10.00±2.80a	10.85±2.44a	14.54±2.93b	15.85±2.38bc	17.38±1.98c	19.92±3.04d
Cyanobacteria	14.54±5.36b	13.23±6.19ab	10.23±4.07a	16.77±4.40b	21.00±5.72c	20.85±6.18c
Gemmatimonadetes	21.15±7.70a	23.69±6.36ab	20.92±3.55a	25.85±4.06ab	20.46±5.25a	28.38±12.77b
Nitrospira	6.23±2.74a	7.38±2.50a	7.00±261a	7.23±2.49a	9.69±1.32b	10.15±1.77b
Armatimonadetes	19.38±4.72b	19.38±3.97b	14.92±3.09a	17.46±5.88ab	26.92±7.16c	28.85±4.81c
BRC1	3.77±1.24a	3.08±1.61a	3.23±0.93a	2.92±1.32a	5.62±2.43b	4.92±2.14b
Chlorobi	1.46±0.88a	2.15±1.14a	1.92±1.19a	1.85±1.34a	1.84±0.90a	1.92±1.44a
WS3	7.69±2.02c	6.00±1.58b	2.15±0.99a	2.53±1.33a	2.85±1.68a	2.62±1.04a
Chlamydiae	4.08±2.87b	2.85±1.77b	1.54±1.56a	0.46±0.66a	0.77±1.24a	0.62±0.87a
OD1	2.69±1.75c	2.23±1.64bc	1.62±1.33ab	1.15±1.21a	1.38±0.65ab	1.38±1.12ab
**Total**	**3292.38±157.16a**	**3186.92±170.78a**	**3299.54±169.64a**	**3144.23±214.65a**	**3516.85±153.0b**	**3472.46±310.75b**

Data present the mean value and standard error. Significant differences among study sites are indicated by alphabetic letters. $P < 0.05$.

Table S3. Soil bacterial relative abundance of dominant (> 0.10%) classes at six study sites

Phylum	Class	SJY-4790	SJY-4480	SJY-4140	SJY-3880	SJY-3490	SJY-3220
Proteobacteria	Alphaproteobacteria	15.85	16.83	20.07	19.87	17.28	18.02
	Betaproteobacteria	7.11	6.41	3.86	3.18	2.78	2.76
	Gammaproteobacteria	10.38	9.15	7.21	4.49	4.32	3.49
	Deltaproteobacteria	1.55	1.55	1.10	1.12	1.60	1.52
Acidobacteria	Acidobacteria_Gp3	0.16	0.09	0.12	0.11	0.22	0.31
	Acidobacteria_Gp4	7.67	8.23	8.52	10.32	11.27	11.78
	Acidobacteria_Gp6	9.74	13.11	10.35	11.77	12.21	11.79
	Acidobacteria_Gp7	1.06	1.03	0.74	0.92	1.16	1.12
	Acidobacteria_Gp10	0.11	0.25	0.53	0.58	0.95	1.12
	Acidobacteria_Gp16	2.10	2.49	2.54	2.43	2.49	2.51
	Acidobacteria_Gp17	0.96	1.26	0.87	0.73	0.56	0.50
	Acidobacteria_Gp22	0.17	0.17	0.02	0.03	0.01	0.00
	Acidobacteria_Gp25	0.17	0.14	0.10	0.11	0.25	0.20
Verrucomicrobia	Verrucomicrobiae	0.53	0.50	0.70	0.35	0.40	0.28
	Spartobacteria	11.41	8.35	7.45	7.16	5.92	5.37
	Subdivision3	0.78	0.54	0.33	0.33	0.58	0.39
	Opitutae	0.16	0.10	0.15	0.14	0.22	0.20
Bacteroidetes	Sphingobacteria	7.87	5.45	4.44	3.79	3.71	3.17
	Flavobacteria	1.66	1.30	0.81	0.31	0.21	0.16
	Bacteroidetes incertaesedis	0.32	0.34	0.70	0.99	0.91	0.75
Actinobacteria	Actinobacteria	9.21	10.50	17.85	19.17	16.96	18.98
Planctomycetes	Planctomycetacia	1.08	1.19	1.35	1.15	1.59	1.36
	Phycisphaerae	0.00	0.00	0.05	0.06	0.09	0.10
Firmicutes	Bacilli	0.60	0.80	1.33	1.14	1.81	1.97
Cyanobacteria /Chloroplast	Chloroplast	0.25	0.11	0.08	0.24	0.20	0.24
	Cyanobacteria	0.10	0.15	0.21	0.27	0.51	0.41
Crenarchaeota	Thermoprotei	0.64	1.32	1.66	1.76	2.00	1.88
Gemmatimonadetes	Gemmatimonadetes	0.43	0.53	0.46	0.68	0.46	0.62
Chloroflexi	Anaerolineae	0.42	0.45	0.27	0.21	0.25	0.21
	Caldilineae	0.03	0.04	0.10	0.06	0.07	0.05
Nitrospira	Nitrospira	0.29	0.37	0.11	0.17	0.28	0.34

Table S4. The soil microbial functional gene relative abundance at different functional gene categories in all six sites

Gene Category	Gene Name	SJY-4790	SJY-4480	SJY-4140	SJY-3880	SJY-3490	SJY-3220
**P cycling**	Ppx	661.08±132.19	702.52±72.62	678.38±180.55	733.85±115.90	766.81±186.92 (*P* = 0.080)	780.61±184.97 (*P* = 0.040)
	Ppk	222.73±47.79	232.42±29.30	225.06±58.34	258.60±52.72	252.84±64.82	266.03±69.70(*P* =0.050)
	phytase	98.26±24.12	99.39±12.17	98.52±25.23	108.77±17.99	111.29±29.72	112.64±25.46
**N cycling** N fixation	nifH	49.61±9.78	53.06±7.57	51.26±14.13	54.48±9.19	56.85±14.49	60.11±13.53 (*P* =0.026)
Nitrification	amoA	299.75±31.19	324.44±40.03	304.25±54.64	340.83±60.08	347.26±82.38 (*P* =0.053)	347.73±82.38 (*P* =0.050)
	hao	121.68±29.39	124.80±21.71	125.63±29.19	135.97±34.62	151.76±39.36 (*P* =0.015)	142.28±27.36 (*P* =0.092)
Denitrification	nosZ	192.77±38.56	212.52±30.76	203.28±61.37	231.99±47.57	241.61±65.53 (*P* =0.025)	246.74±71.44 (*P* =0.014)
	norB	54.39±8.36	62.16±7.92	56.58±13.41	59.76±8.46	64.60±13.68 (*P* =0.023)	64.73±13.41 (*P* =0.021)
	nirS	84.02±15.81	89.75±11.60	84.83±22.27	93.35±14.45	97.80±22.50 (*P* =0.070)	94.16±24.30 (*P* =0.180)
	nirK	200.10±31.21	223.61±24.43	208.58±49.32	233.06±41.37	246.72±56.05 (*P* =0.014)	244.15±67.15 (*P* =0.020)
	narG	33.42±5.87	35.41±3.68	33.40±7.35	35.62±3.75	35.35±6.33	35.96±6.57
Dissimilarory N reduction to ammonium	napA	57.26±6.42	60.49±8.26	55.39±10.82	57.37±8.08	63.60±12.80	62.86±13.21
	nifA	91.79±14.32	98.93±11.78	90.77±22.45	100.95±16.61	103.66±25.22	101.70±23.31
Ammonification	ureC	414.38±65.04	420.75±45.89	403.54±87.10	439.11±60.98	447.83±92.62	449.66±84.60
	gdh	374.03±40.28	431.62±49.96 (P=0.049)	380.54±65.89	422.63±73.81	437.01±101.39 (*P* =0.032)	428.38±89.65 (*P* =0.063)
Assimilatory N reduction	NirB	174.24±25.23	198.55±24.76	183.64±44.50	205.37±39.34	218.33±52.38 (*P* =0.010)	219.67±57.85 (*P* =0.008)
	nirA	165.93±27.17	179.39±24.70	166.54±41.70	190.52±33.95	199.56±50.66 (*P* =0.032)	195.80±48.77 (*P* =0.055)
	NirR	176.38±28.04	192.18±25.55	177.43±44.06	201.06±33.98	212.77±52.90 (*P* =0.026)	208.23±51.79 (*P* =0.051)
	nasA	34.58±6.97	35.93±3.75	34.42±8.04	37.98±5.41	35.73±7.74	38.34±6.94

Anammox	hzo	69.02±8.80	78.26±11.90	68.35±15.06	73.34±14.28	86.16±19.14 (*P* =0.004)	81.15±17.19 (*P* =0.040)
**Carbon cycling**	AmyA	2811.01±375.5	3028.88±409.2	2764.56±385.2	3109.61±651.6	2997.04±787.3	3049.70±609.50
Starch degradation	pulA	125.61±12.60	136.13±15.33	125.65±19.57	137.88±19.09	138.95±29.80	140.86±30.13 (*P* =0.083)
Hemicellulose degradation	ara	290.19±34.56	310.25±39.96	285.30±57.89	320.63±44.59	329.95±70.36 (*P* =0.065)	323.23±67.22
	Mannanase	142.17±28.22	145.40±17.01	145.06±37.26	153.86±22.03	161.88±39.85	163.46±35.24 (*P* =0.085)
	xylanase	32.58±5.25	35.09±4.08	32.06±7.39	37.40±6.82	37.48±8.39 (*P* =0.092)	38.99±10.28 (*P* =0.028)
	xylA	80.92±13.50	87.83±13.13	78.87±18.41	91.65±19.71	92.49±21.92	94.08±24.95 (*P* =0.083)
Cellulose degradation	Endoglucanase	151.93±21.53	156.41±20.62	149.15±30.07	163.77±24.87	172.01±36.26 (*P* =0.082)	166.82±36.62
	Exoglucanase	42.96±8.87	46.53±7.95	44.40±13.82	50.16±10.62	52.67±13.53 (*P* =0.047)	51.77±16.44 (*P* =0.071)
Chitin degradation	Exochitinase	28.22±3.77	30.54±3.73	28.86±5.96	31.57±4.02	33.54±5.98 (*P* =0.011)	32.03±6.90 (*P* =0.067)
	Acetylglucosaminidase	400.14±62.66	425.35±70.70	386.16±90.39	438.70±74.67	442.31±100.10	460.30±119.25 (*P* =0.087)
	Endochitinase	211.30±29.16	221.87±22.66	209.09±38.81	234.94±31.13	232.07±43.96	234.57±44.92
Aromatics degradation	vdh	35.67±4.03	38.80±5.38	35.60±8.26	39.50±5.65	42.60±9.48 (*P* =0.018)	40.68±9.07 (*P* =0.084)
	vanA	191.97±27.03	199.41±25.64	187.96±40.02	212.12±31.99	215.69±41.85 (*P* =0.091)	213.65±41.63
Lignin degradation	Phenol oxidase	193.93±23.51	200.03±19.45	193.03±38.17	209.88±28.79	216.10±44.17	213.38±40.38
	mnp	22.77±4.07	24.49±2.50	22.62±4.65	25.38±3.86	25.51±5.00	24.54±5.82
Carbon fixation	pcc	397.02±63.82	420.52±62.30	392.57±91.78	455.61±86.79	452.79±102.02	462.47±105.56 (*P* =0.059)
	Rubisco	173.54±23.69	188.13±27.03	169.95±34.14	190.12±33.73	198.40±45.13 (*P* =0.084)	192.92±47.36
	FTHFS	233.40±32.40	257.34±34.45	228.10±49.67	263.59±41.15	276.88±63.76 (*P* =0.027)	269.80±63.53 (*P* =0.063)
Methane metabolism	pmoA	33.70±4.26	38.50±5.56	33.20±7.98	39.57±7.15	40.79±10.03 (*P* =0.024)	40.80±10.29 (*P* =0.024)
	mcrA	56.94±15.29	61.34±13.56	59.81±20.43	72.95±21.91	74.99±25.05 (*P* =0.033)	75.10±27.35 (*P* =0.032)

Table S5. Pearson correlation between plant and bacterial alpha diversity and individual environmental factors.

**Environmental Factors**	**Plant Shannon Index**		**Bacteria Shannon Index**	
	**r**	***P***	**r**	***P***
Site Elevation	0.128	0.265	-0.532	< 0.001
Mean annual temperature	-0.081	0.483	0.549	< 0.001
Mean annual precipitation	0.109	0.343	-0.471	< 0.001
Soil moisture	0.188	0.099	-0.464	< 0.001
Soil pH	-0.097	0.399	0.477	< 0.001
Soil organic carbon	0.082	0.476	-0.469	< 0.001
Soil total nitrogen	0.115	0.316	-0.445	< 0.001
Soil total phosphorus	-0.009	0.936	-0.353	0.002
Soil available nitrogen	0.116	0.314	-0.449	< 0.001
Rapid available phosphorus	-0.001	0.992	-0.278	0.014
NH_4^+-N	0.001	0.992	-0.304	0.006
NO_3^--N	0.152	0.183	0.086	0.456
Bacteria / Plant Shannon index	-0.147	0.198	-0.147	0.198

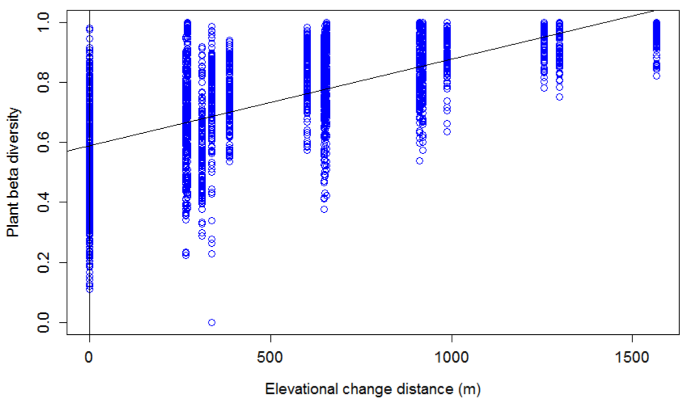

(**a**)

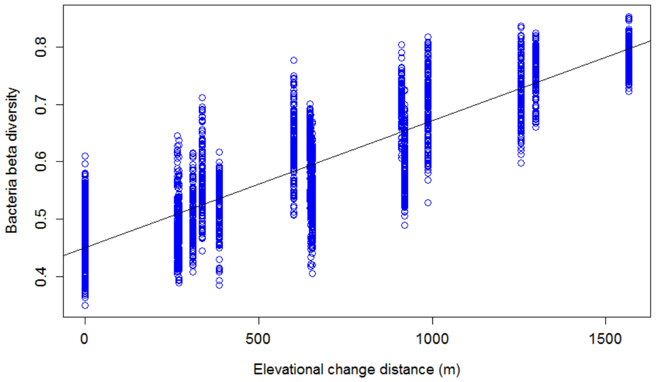

(**b**)

Figure S1. The relationships between plant beta diversity (**a**) (r= 0.696, *P* = 0.001) and soil bacterial beta diversity (**b**) (r= 0.865, *P* = 0.001) and change elevation distance.
